# PURE: A webserver for the prediction of domains in unassigned regions in proteins

**DOI:** 10.1186/1471-2105-9-281

**Published:** 2008-06-14

**Authors:** Chilamakuri CS Reddy, Khader Shameer, Bernard O Offmann, Ramanathan Sowdhamini

**Affiliations:** 1 National Centre for Biological Sciences, Tata Institute of Fundamental Research, GKVK Campus, Bellary Road, Bangalore 560 065, India; 2 Laboratoire de Biochimie et Genetique Moleculaire, Universite de La Reunion, 15 avenue Rene Cassin, BP 7151, 97715 Saint Denis Messag Cedex 09, La Reunion, France

## Abstract

**Background:**

Protein domains are the structural and functional units of proteins. The ability to parse proteins into different domains is important for effective classification, understanding of protein structure, function, and evolution and is hence biologically relevant. Several computational methods are available to identify domains in the sequence. Domain finding algorithms often employ stringent thresholds to recognize sequence domains. Identification of additional domains can be tedious involving intense computation and manual intervention but can lead to better understanding of overall biological function. In this context, the problem of identifying new domains in the unassigned regions of a protein sequence assumes a crucial importance.

**Results:**

We had earlier demonstrated that accumulation of domain information of sequence homologues can substantially aid prediction of new domains. In this paper, we propose a computationally intensive, multi-step bioinformatics protocol as a web server named as **PURE **(**P**rediction of **U**nassigned **RE**gions in proteins) for the detailed examination of stretches of unassigned regions in proteins. Query sequence is processed using different automated filtering steps based on length, presence of coiled-coil regions, transmembrane regions, homologous sequences and percentage of secondary structure content. Later, the filtered sequence segments and their sequence homologues are fed to PSI-BLAST, cd-hit and Hmmpfam. Data from the various programs are integrated and information regarding the probable domains predicted from the sequence is reported.

**Conclusion:**

We have implemented PURE protocol as a web server for rapid and comprehensive analysis of unassigned regions in the proteins. This server integrates data from different programs and provides information about the domains encoded in the unassigned regions.

## Background

Protein domains are the structural and functional units of proteins and represent one of the most useful levels to understand protein function. Analysis of proteins at the level of domain families has had a profound impact on the study of individual proteins. Protein domain discovery using various computational approaches has been progressing steadily over the past 35 years [1]. They can be defined using multiple criteria, or combinations of criteria, including evolutionary conservation, discrete functionality, and the ability to fold independently [2]. The identification of protein domains within a polypeptide chain can be achieved in several ways. Methods applied by classification databases such as the Dali domain dictionary [3], CATH [4], SCOP [5], DIAL [6] employ structural data to locate and assign domains. Identification of domains at the sequence level most often relies on the detection of global and local sequence alignments between a given target sequence and domain sequences found in databases such as Pfam [7]. However, difficulties in elucidating the domain content of a given sequence arise when searching the target sequence against sequence databases resulting in a lack of significant matches. For example, *Mycoplasma genitalium *is a small genome with 483 proteins but only 386 protein sequences have known Pfam hits with 56% residue coverage. In such situations, there is a need to further explore other methods for domain assignment from sequence. Though, similar approaches of integrating multiple, sensitive database searches to detect distant homologues has been reported as a successful method to establish remote homology [8], we have recently shown that it is possible to enhance prediction of domains by 25% through indirect connections, namely consulting the domain architecture of sequence homologues [9]. In this paper, we report the availability of a bioinformatics protocol, as a web server called **PURE **(**P**rediction of **u**nassigned **re**gions in proteins), which will enhance the domain predictions. PURE protocol utilizes the concept of intermediate sequence search (ISS) [10] to assign functional domain to a given unassigned region with the help of connecting sequences. Indirect connections between the query and distantly related domain is established through a powerful procedure using PSI-BLAST hits which are individually routed through a rigorous Hmmpfam search against Pfam database [11].

## Implementation

### PURE server: description and features

**Figure 1:**
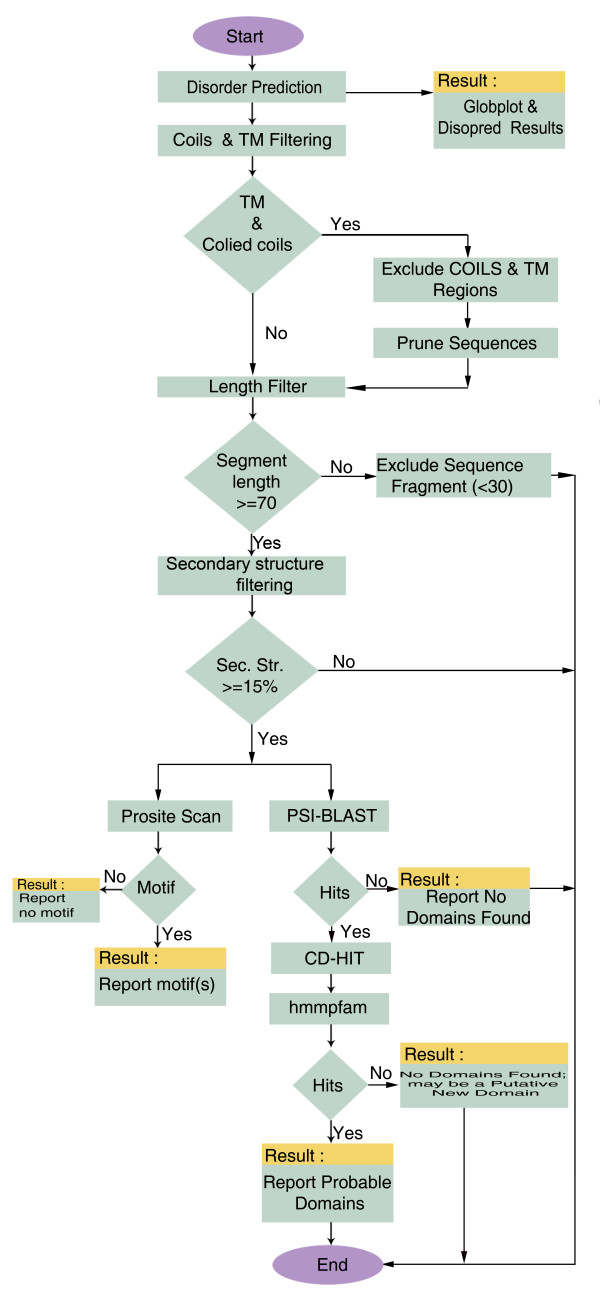
PURE protocol flowchart.

PURE server processes the query sequence in FASTA format using a computationally intensive bioinformatics protocol. A detailed flow-chart of the protocol is provided in Figure 1. The web interface is developed in HTML and Java script. The wrapper scripts for external programs like Globplot [12], Disopred2 [13] Pepcoils [14], Tmap [14], psipred [15], Scanprosite [16], PSI-BLAST [17], cd-hit [18] and Hmmpfam [19], while core programs that integrate results, CGI programs and automated e-mail programs are coded in Perl. Multiple alignment visualization of PSI-BLAST output is enabled using Mview [20]. The graphics that integrate the results are generated using Bio::graphics module from Bioperl [21].

### Input options

**Figure 2:**
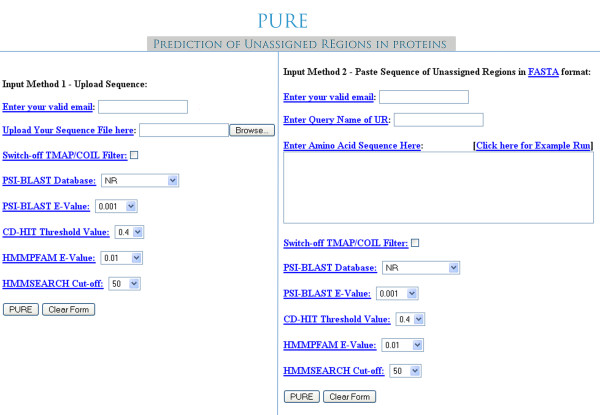
Screenshot of input options from PURE server.

PURE server is designed to accept a single sequence in FASTA format at a time (Figure 2). Users can submit the sequence as a file using the upload option or copy paste option. Users should also submit a valid, non-commercial email address to the server. PURE server will send the result URL to the email address. Options are provided to select E-value for the PSI-BLAST search and Hmmpfam [16] search against various sequence databases like NCBI-NR [22] and SwissProt [23]. Options are also provided to change the clustered sequence space obtained from PSI-BLAST search using different threshold value for cd-hit. User can also choose the filtering option.

### Output details

**Figure 3:**
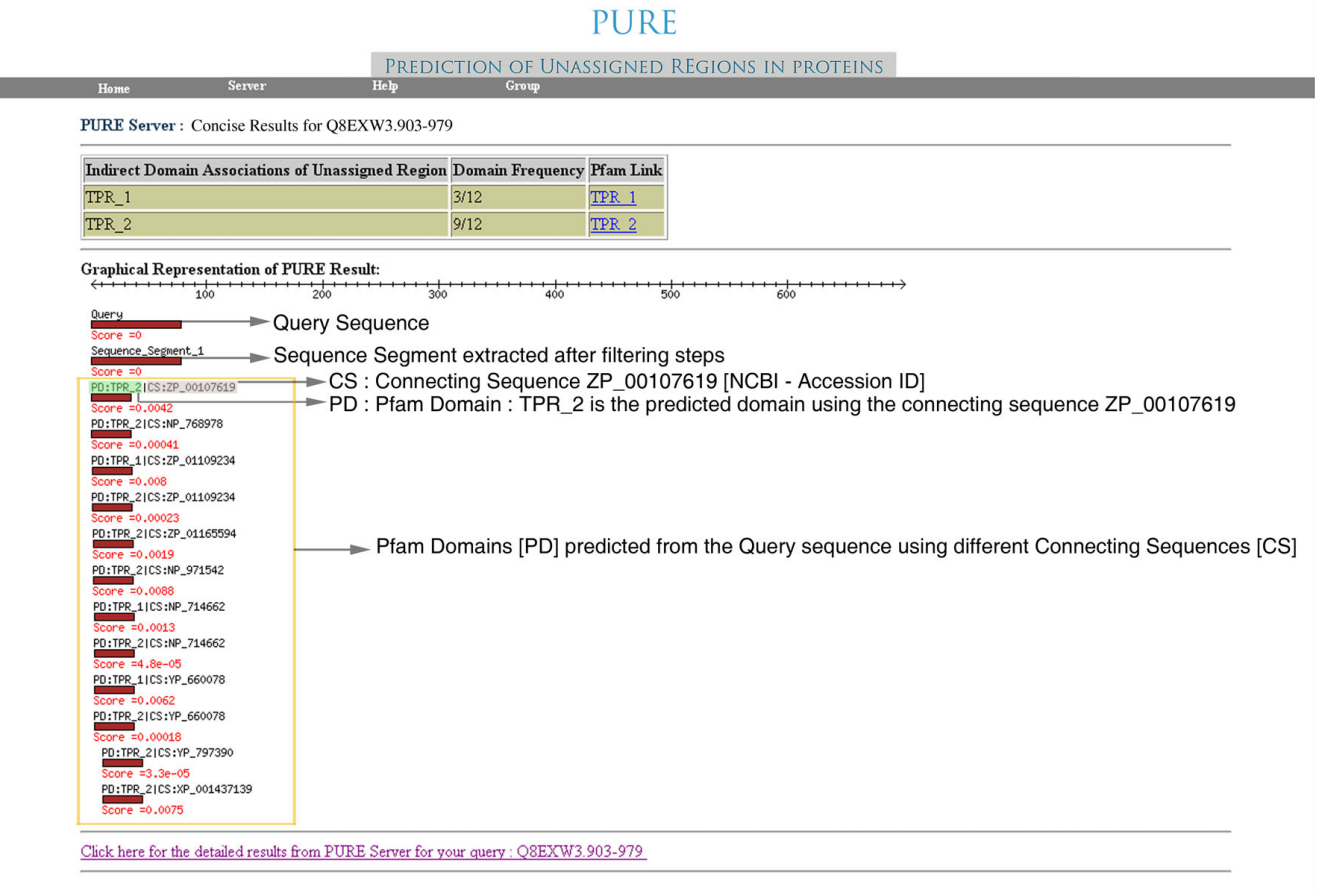
Screenshot of concise output from PURE server.

PURE Server examines unassigned regions for the presence of disordered regions, coiled coils, transmembrane helices, appropriate extent of predicted secondary structural content and presence of homologous sequences before the assignment of probable structural domains. These are also provided as links to the URL where the output is stored in. Output can be mainly divided into two:

A. Consensus output: provides a summary of the overall results (Figure 3). Consensus result page provides a complete overview of the PURE results for the query sequence. The First table in the Consensus result provides the details about the domain assigned, if any, using PURE protocols. This table provides the name of the domain associated to the unassigned region, domain frequency and the direct link to the Pfam database [7] of the identified domain. The table is followed by a graphical representation of PURE Results indicating domain boundaries and the region of similarity between the unassigned region and the associated Pfam domains. This figure is dynamically generated after processing complete results from PURE run for the query sequence. Further, the Bio::graphics [19] based picture provides a complete overview of the query sequence (query), transmembrane regions (Tmap), coiled coil regions (Pepcoil), extracted sequence segments (sequence_segments) after removal of transmembrane and coiled coil regions from the query sequence and the domains assigned by PURE. Apart from the above mentioned information, consensus results page also provides a link to the detailed results page of the PURE results.

**Figure 4:**
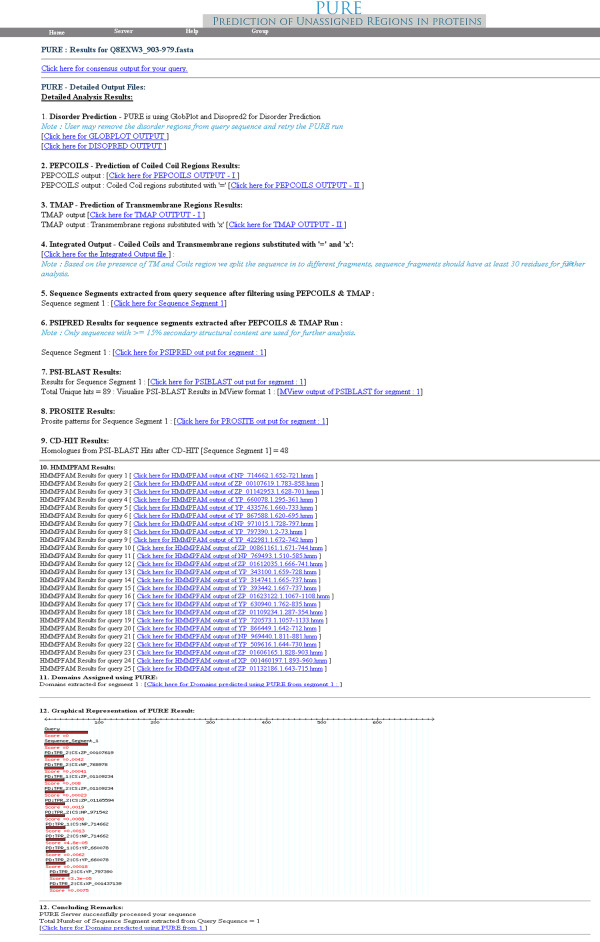
Screenshot of detailed results from PURE server.

B. Detailed output: This output is divided into 10 sections for a successful PURE run. Each of the files provided in detailed output gives the background details about the final result file (Figure 4).

1. Disorder prediction: This section shows the predicted disordered regions in the query sequence. We have employed Globplot [12] and Disopred2 [13] programs for the prediction of disordered regions in the protein sequence.

2. Pepcoils results: This file is generated for the prediction of coiled coils by Pepcoils (EMBOSS) [14] program that works around COILS [24] algorithm. Details about the coiled coils identified from the query sequence are available in this file. Another file with the query sequence parsed for coiled coil regions (such regions are substituted with '=') is also provided.

3. Tmap results: This file is generated to record probable transmembrane helices in the query sequence as identified by Tmap (EMBOSS) [14] program. Another file with transmembrane regions identified from query sequence substituted with 'x' is also provided for better understanding of the presence of transmembrane regions.

4. Integrated filter results: Both COILS and Tmap files are processed using a Perl program. This program integrates the query sequence to provide a modified query sequence file, with coiled coils and transmembrane regions substituted with '=' and 'x' respectively. By default, such regions are not considered for further analysis. Further, the sequence is split into segments based on the presence of transmembrane regions and coiled coil regions. Only sequence segments having ≥ 30 residues are considered for further analysis, in order to avoid spurious hits in subsequent PSI-BLAST jobs.

5. Examine the sequence segments extracted from query sequence after filtering using Pepcoils and Tmap. Sequence is split into different fragments based on the presence of coiled coil and transmembrane regions, each fragment should have at least 30 residues to be considered for further analysis.

6. Psipred results: We have used psipred [15] program for secondary structure prediction, sequence segments with ≥ 15% secondary structural content considered for further analysis.

7. PSI-BLAST result file: Sequence segments which passed the filtering criteria above are fed to PSI-BLAST [17] for similarity search. Mview based visualization of PSI-BLAST results are also provided for better insight into the PSI-BLAST results.

8. Scanprosite results: Prosite [16] scan results are supplied as a supplement to our method. Scanprosite scans query sequence for the occurrence of patterns, profiles and rules (motifs) stored in the Prosite database [25].

9. Hmmpfam Results: The results obtained by running Hmmpfam on homologous sequences and the assignment of Pfam domains for homologues are provided here. Only representative sequences, as recognized after clustering by cd-hit [18] are considered for the search for domains.

10. Domains assigned using PURE: This is the major output file that integrates the PSI-BLAST and Hmmpfam search output and provide the probable domains identified using PURE protocol for the unassigned regions in the query. Bio::graphics [21] based image is provided for the better overview of the detailed result.

11. Concluding remarks: The concluding mark is derived at the end of each run after analyzing all the files that are generated. Various output possibilities and concluding remarks are discussed in Table 1.

**Table 1:** Concluding remarks from PURE server based on various outputs

**Case**	Output details	Concluding remarks
**1**	Output Files : Globplot, Disopred2 and Pepcoils or Globplot, Disopred2 and Tmap or Globplot,, Disopred2, Pepcoils and Tmap.	Query sequence is divided into sequence segments based on Pepcoils and Tmap output. After excluding the sequence portions encoded by transmembrane and coiled-coils, the unassigned region could be associated with coiled coils or TM helices and the rest of the unassigned regions are insignificantly small and therefore structure association terminates.
**2**	Output Files : Pepcoils, Tmap and psipred	psipred results suggest very little secondary structure, therefore the unassigned region is to be viewed as unstructured and procedure terminates.
**3**	Output Files : Pepcoils, Tmap, psipred and PSI-LAST hits (less homologues)	PSIBLAST did not provide enough homologues and terminates. Here, the user could relax thresholds or choose a different database and retry. If there is enough secondary structural content, this unassigned region could be a potential new domain that is somewhat species-specific and hence not evolutionarily conserved.
**4**	Output files: Pepcoils, Tmap, psipred, PSI-BLAST and Hmmpfam out put (no hits in Hmmpfam search)	The indirect HMM runs and the consensus did not yield any hit to a Pfam domain. The user can relax cd-hit and get more homologues included in the search or relax the HMM threshold and retry. Or else, if there is enough predicted secondary structural content and the unassigned region is evolutionarily conserved, this could point to a potential new and novel domain.
**5**	Output files: Pepcoils, Tmap, psipred, PSI-BLAST and Hmmpfam out put (hits in Hmmpfam search with partial domain assignment)	The indirect HMM runs point to partial assignment to a pre-existing Pfam domain family. This could be due to suboptimal or spurious alignment or point to the presence of discontinuous domains in the query.
**6**	Output files: Pepcoils, Tmap, psipred, PSI-BLAST and Hmmpfam out put (hits in Hmmpfam search with complete domain assignment)	The indirect HMM runs point to assignment to a pre-existing Pfam domain family through one or more homologues. This could be due to distant relationships where the homologues act as intermediates or due to borderline E-values. New connection to an old domain!
**7**	Output files: Pepcoils, Tmap, psipred, PSI-BLAST and Hmmpfam out put (hits in Hmmpfam search against UR itself)	Direct HMM assignment to a pre-existing Pfam family. This could be due to newer Pfam domain entries or slight differences in thresholds to E-values. Connection to a fairly new domain.

## Results and Discussion

In cases where the PURE server is not effective with default parameters, the user can manoeuvre the search directions by changing certain parameters. However, the minimum length of 30 residues for an unassigned region (UR) is not recommended to go lower, in order to avoid sub-optimal alignments and false positives. By default, unassigned region will be split whenever transmembrane helices (TM) or coiled coils regions are encountered; however, there is a possibility to override this option. Likewise, the E-value thresholds of PSI-BLAST runs and cd-hit thresholds for representative homologous sequences can be modified according to the choice of the user. It is also possible to increase the number of homologues identified and simultaneously decrease the cd-hit clustering threshold so that fewer representatives are examined for the domain architecture. This strategy is recommended to improve the sampling of sequence space.

We have tested the reliability of the PURE algorithm as well as the performance of the server using various datasets. PURE protocol was successfully employed to annotate 67 adenylyl cyclase proteins with unassigned regions [24] and in *Mycoplasma gallisepticum *genome analysis where 82 new domains are added to 72 proteins out of which 48 proteins are earlier completely unassigned (unpublished data).

## Conclusion

Due to advancement in high-throughput techniques, sequence data are generating at a rapid pace, but the biochemical validation of available sequences is still a challenge. Efficient computational methods can be employed to overcome the lacunae in high-throughput sequence data generation and function annotation. PURE can be used as an efficient and computationally intensive protocol to successfully annotate unassigned regions in sequences.

## Availability and requirements

Project name: PURE – Prediction of domains in the protein unassigned regions

Project home page: [26]

A sample consensus page, with above explained features, is available: [27]

Pre-computed results for selected unassigned regions from earlier analysis [9] are available: [28]

Operating system(s): Platform independent (web server)

Programming language: HTML, Perl, CGI, Java script

License: Free for academics, Authorization license needed for commercial usage (Please contact the corresponding author for more details)

Any restrictions to use by non-academics: license needed

## Authors' contributions

RS and BOO conceived and helped in the implementation of the webserver idea. CCSR and KS have developed the scripts and tools for the webserver. CCSR and KS wrote the manuscript; RS and BOO provided critical inputs to improve the manuscript.

## References

[B1] Copley RR, Doerks T, Letunic I, Bork P (2002). Protein domain analysis in the era of complete genomes. FEBS Lett.

[B2] Holm L, Sander C (1994). Parser for protein folding units. Proteins.

[B3] Dietmann S, Holm L (2001). Identification of homology in protein structure classification. Nat Struct Biol.

[B4] Orengo CA, Michie AD, Jones S, Jones DT, Swindells MB, Thornton JM (1997). CATH--a hierarchic classification of protein domain structures. Structure.

[B5] Murzin AG, Brenner SE, Hubbard T, Chothia C (1995). SCOP: a structural classification of proteins database for the investigation of sequences and structures. J Mol Biol.

[B6] Sowdhamini R, Blundell TL (1995). An automatic method involving cluster analysis of secondary structures for the identification of domains in proteins. Protein Sci.

[B7] Sonnhammer EL, Eddy SR, Birney E, Bateman A, Durbin R (1998). Pfam: multiple sequence alignments and HMM-profiles of protein domains. Nucleic Acids Res.

[B8] Salamov AA, Suwa M, Orengo CA, Swindells MB (1999). Combining sensitive database searches with multiple intermediates to detect distant homologues. Protein Eng.

[B9] Reddy CS, Manonmani A, Babu M, Sowdhamini R (2006). Enhanced structure prediction of gene products containing class III adenylyl cyclase domains. In Silico Biol.

[B10] Park J, Teichmann SA, Hubbard T, Chothia C (1997). Intermediate sequences increase the detection of homology between sequences. J Mol Biol.

[B11] Bateman A, Coin L, Durbin R, Finn RD, Hollich V, Griffiths-Jones S, Khanna A, Marshall M, Moxon S, Sonnhammer EL, Studholme DJ, Yeats C, Eddy SR (2004). The Pfam protein families database. Nucleic Acids Res.

[B12] Linding R, Russell RB, Neduva V, Gibson TJ (2003). GlobPlot: Exploring protein sequences for globularity and disorder. Nucleic Acids Res.

[B13] Ward JJ, Sodhi JS, McGuffin LJ, Buxton BF, Jones DT (2004). Prediction and functional analysis of native disorder in proteins from the three kingdoms of life. J Mol Biol.

[B14] Rice P, Longden I, Bleasby A (2000). EMBOSS: the European Molecular Biology Open Software Suite. Trends Genet.

[B15] McGuffin LJ, Bryson K, Jones DT (2000). The PSIPRED protein structure prediction server. Bioinformatics.

[B16] de Castro E, Sigrist CJ, Gattiker A, Bulliard V, Langendijk-Genevaux PS, Gasteiger E, Bairoch A, Hulo N (2006). ScanProsite: detection of PROSITE signature matches and ProRule-associated functional and structural residues in proteins. Nucleic Acids Res.

[B17] Altschul SF, Madden TL, Schaffer AA, Zhang J, Zhang Z, Miller W, Lipman DJ (1997). Gapped BLAST and PSI-BLAST: a new generation of protein database search programs. Nucleic Acids Res.

[B18] Li W, Godzik A (2006). Cd-hit: a fast program for clustering and comparing large sets of protein or nucleotide sequences. Bioinformatics.

[B19] Eddy SR (1998). Profile hidden Markov models. Bioinformatics.

[B20] Brown NP, Leroy C, Sander C (1998). MView: a web-compatible database search or multiple alignment viewer. Bioinformatics.

[B21] Stajich JE, Block D, Boulez K, Brenner SE, Chervitz SA, Dagdigian C, Fuellen G, Gilbert JG, Korf I, Lapp H, Lehvaslaiho H, Matsalla C, Mungall CJ, Osborne BI, Pocock MR, Schattner P, Senger M, Stein LD, Stupka E, Wilkinson MD, Birney E (2002). The Bioperl toolkit: Perl modules for the life sciences. Genome Res.

[B22] Wheeler DL, Barrett T, Benson DA, Bryant SH, Canese K, Chetvernin V, Church DM, DiCuccio M, Edgar R, Federhen S, Geer LY, Kapustin Y, Khovayko O, Landsman D, Lipman DJ, Madden TL, Maglott DR, Ostell J, Miller V, Pruitt KD, Schuler GD, Sequeira E, Sherry ST, Sirotkin K, Souvorov A, Starchenko G, Tatusov RL, Tatusova TA, Wagner L, Yaschenko E (2007). Database resources of the National Center for Biotechnology Information. Nucleic Acids Res.

[B23] Bairoch A, Apweiler R (2000). The SWISS-PROT protein sequence database and its supplement TrEMBL in 2000. Nucleic Acids Res.

[B24] Lupas A, Van Dyke M, Stock J (1991). Predicting coiled coils from protein sequences. Science.

[B25] Hulo N, Bairoch A, Bulliard V, Cerutti L, De Castro E, Langendijk-Genevaux PS, Pagni M, Sigrist CJ (2006). The PROSITE database. Nucleic Acids Res.

[B26] PURE server home page. http://caps.ncbs.res.in/PURE.

[B27] Sample consensus page. http://caps.ncbs.res.in/PURE/PURE_Results/PURE_Consensus/TueAug21095436IST2007_consensus.html.

[B28] Pre-computed results. http://caps.ncbs.res.in/PURE/example_result.html.

